# Quantifying the threat of extinction from Muller's ratchet in the diploid Amazon molly (*Poecilia formosa*)

**DOI:** 10.1186/1471-2148-8-88

**Published:** 2008-03-19

**Authors:** Laurence Loewe, Dunja K Lamatsch

**Affiliations:** 1 Institute of Evolutionary Biology, School of Biological Sciences, University of Edinburgh, Ashworth Laboratories, King's Buildings, Edinburgh EH9 3JT, UK; 2 Centre for Systems Biology Edinburgh, School of Biological Sciences, University of Edinburgh, Darwin Building, King's Buildings, Edinburgh EH9 3JU, UK; 3 Universität Würzburg, Institute of Physiological Chemistry I, Biocenter, Würzburg, 97074 Würzburg, Germany; 4 Freshwater Biology, Royal Belgian Institute of Natural Sciences, Vautierstraat 29, B – 1000 Brussels, Belgium; 5 University of Sheffield, Department of Animal and Plant Sciences, Alfred Denny Building, Western Bank, Sheffield, S10 2TN, UK; 6 Austrian Academy of Sciences, Institute for Limnology, Mondseestrasse 9, 5310 Mondsee, Austria

## Abstract

**Background:**

The Amazon molly (*Poecilia formosa*) is a small unisexual fish that has been suspected of being threatened by extinction from the stochastic accumulation of slightly deleterious mutations that is caused by Muller's ratchet in non-recombining populations. However, no detailed quantification of the extent of this threat is available.

**Results:**

Here we quantify genomic decay in this fish by using a simple model of Muller's ratchet with the most realistic parameter combinations available employing the evolution@home global computing system. We also describe simple extensions of the standard model of Muller's ratchet that allow us to deal with selfing diploids, triploids and mitotic recombination. We show that Muller's ratchet creates a threat of extinction for the Amazon molly for many biologically realistic parameter combinations. In most cases, extinction is expected to occur within a time frame that is less than previous estimates of the age of the species, leading to a genomic decay paradox.

**Conclusion:**

How then does the Amazon molly survive? Several biological processes could individually or in combination solve this genomic decay paradox, including paternal leakage of undamaged DNA from sexual sister species, compensatory mutations and many others. More research is needed to quantify the contribution of these potential solutions towards the survival of the Amazon molly and other (ancient) asexual species.

## Background

It is a general observation that asexual lineages do not last over very long periods of time, but the precise reasons for this are less clear [1]. Ancient asexuals are the rare exceptions to this rule and it is of considerable biological interest to know what mechanisms allow them to survive for so long. They must have found a way to overcome the long-term fitness-degrading consequences of genetic processes like Muller's ratchet and/or ecological processes like Red-Queen dynamics [1-6]. Muller's ratchet describes the long-term accumulation of slightly deleterious mutations in asexual populations and has been suggested as a key mechanism for the extinction of asexual species on the long term [3,7,8]. Unfortunately most of these predictions remain at a stage of verbal argument [1], making it very difficult to rule out that Muller's ratchet may not have had enough time to cause extinction. As the behavior of Muller's ratchet can be very sensitive to model parameters [7], realistic values need to be used to predict the consequences of mutation accumulation for a given ancient asexual species. We employ a simple null hypothesis [see [7]] for testing the threat of extinction from Muller's ratchet in the unisexual fish *Poecilia formosa*, the Amazon molly.

### Overcoming the lack of quantification

Quantifications of the threat of extinction from Muller's ratchet are often not trivial theoretical work that requires either challenging mathematics [9] or complex computer simulations [7] or both [7]. Therefore some adopt the pragmatic approach that any system with no recombination and a potential for appreciable slightly deleterious mutation rates could be driven to extinction by Muller's ratchet [3]. Such arguments frequently overlook the fact that the particular combination of parameters in that species might not be expected to lead to extinction within the known time of its existence, even if Muller's ratchet is clearly operating [1,3,7]. Thus statements about the evolutionary short lives of asexuals are often less quantitative than would be desirable [1]. In other words there may not be a genomic decay paradox that calls for any special solutions [7]. We advocate the use of a simple model for predicting extinction times caused by Muller's ratchet in order to make current discussions about ancient asexuals more quantitative [1,7], even if that model cannot capture the full complexity of our study species and therefore only leads to tentative predictions. We believe that small steps in model development will allow future models to benefit from the experiences with simpler models. Hence our use of a simple model of Muller's ratchet that ignores all complications like potential Red Queen dynamics that might accelerate the rate of Muller's ratchet (see discussion below and [6]). We focus on testing the null hypothesis that Muller's ratchet could not have led to extinction in a given time frame, as described by Loewe [7]. While this is an important advance over the purely verbal stage, we want to encourage future work to model the various processes that increase or decrease the predicted speed of genomic decay. We also want to encourage more empirical work to establish the precise values of parameters in these models. Such work is needed for other asexual species as well.

### The Amazon molly and Muller's ratchet

The fish *Poecilia formosa *was the first unisexual vertebrate that was discovered [10]. This all-female species resulted from hybridization between relatives of *Poecilia mexicana *and *Poecilia latipinna *[11,12] that probably happened between 40,000 and 100,000 years ago (see section 'Age ...' below and [11,12]). Reproduction normally occurs by sperm-dependent parthenogenesis, i.e. diploid eggs are produced, which need to be activated for embryonic development by sperm of closely related species. It has been argued that paternal leakage, leading to the expression of paternal genes, plays a pivotal role to stop Muller's ratchet [13] that otherwise would have driven the species to extinction in less time than its current estimates of existence [11]. Paternal leakage and other processes that may slow down genomic decay are discussed below. We want to determine if these processes are necessary to explain the survival of this fish into our times.

### Habitat and population structure

The Amazon molly is a small fish (3 – 7 cm) that lives in a rather limited range from the Nueces River in south-east Texas southward to the mouth of the Rio Tuxpan, north of the Sierra del Abra in Mexico. All these river systems flow from west to east and have no connection other than the sea. The population on such a large scale may have some structure, as populations from south Texas, for example, have no reasonable connection with those in the Río Purificación. However, as the Amazon molly tolerates marine conditions [14], migration cannot be entirely excluded. A study of *F*_*ST *_in subpopulations that span a distance of about 100 km in the same river system did not find significant population subdivision [15]. Some simple models of population subdivision do not affect the effective population size *N*_*e *_and probabilities of the fixation [such as some island models [16,17]]. However, more realistic models of population structure that allow for extinctions and recolonizations can have a substantial impact on deleterious mutation accumulation [18,19]. To simplify our theoretical treatment, we will assume that the whole species has no substructure that is not already accounted for by our assumed *N*_*e*_.

### Genetics

The Amazon molly reproduces gynogenetically, i.e. its eggs contain an unreduced set of chromosomes, that need the sperm of one of the sister species *Poecilia mexicana *or *Poecilia latipinna *as a mechanical trigger to start development [20-22]. Usually, only the diploid set of maternal genes is expressed and the paternal genome is expelled. However, occasionally, the paternal genome remains, giving rise to a triploid clone, that reproduces as a triploid gynogen [15,23]. In other cases, only traces of the paternal genome (so-called micro-chromosomes) escape the enzymatic machinery that clears the egg from the nucleus that arrived with the sperm [24]. In all cases, the full diploid or triploid set of chromosomes (with or without micro-chromosomes [24,25]) is clonally passed on to offspring, without an obvious opportunity for recombination [26]. Based on this lack of recombination it was hypothesized that Muller's ratchet should have driven the Amazon molly to extinction within the presumed time of its existence, unless processes like paternal leakage would stop genomic decay [13].

### Aims

Here we aim to quantify the verbally predicted effects of Muller's ratchet in order to see, whether there really is a genomic decay paradox as defined by Loewe [7] that calls for an explanation. Results show that indeed a range of realistic parameter combinations should have led to the extinction of Amazon molly within the time of its presumed existence.

## Results

We quantified the rate of mutation accumulation due to Muller's ratchet using the best available analytical approximations [9,27,28] and globally distributed individual-based simulations run by Simulator005r6 of evolution@home [7,29-31] assuming our estimates of the most realistic parameter combinations. To this end we used the standard null model of Muller's ratchet described elsewhere [7] and extended it to accommodate the slowdown in fitness decay that can be caused by polyploidy and mitotic recombination. We employ the U-shaped plot of extinction times against selection coefficients to allow easy visualization of situations that lead to a genomic decay paradox. The frequency of these situations can be measured by specifying the range of critical selection coefficients *s*_*c *_that are defined by the prediction of corresponding extinction times that are below *T*_*age*_, the presumed age of asexuality in an evolutionary line. For more detailed explanations of this plot see Loewe [7].

**Figure 1:**
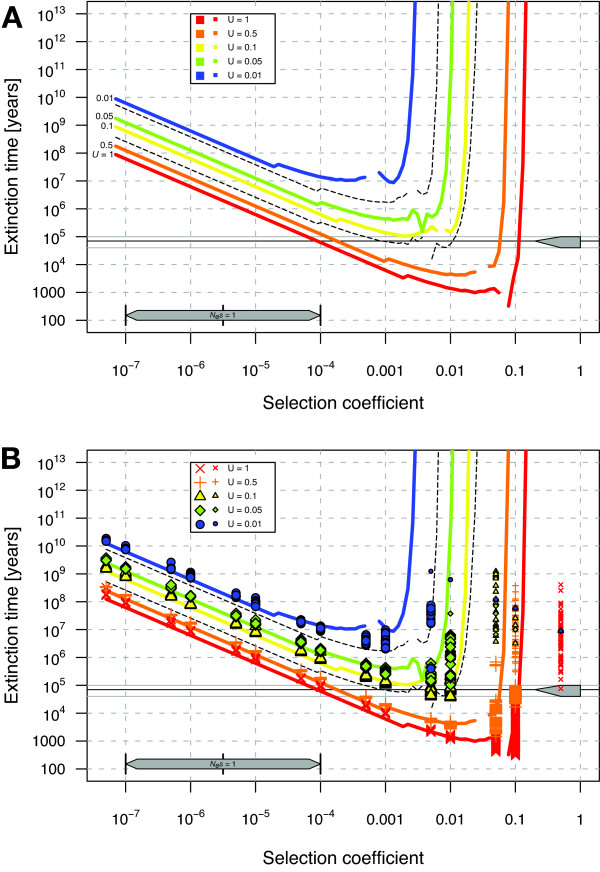
**Predicted extinction times of the Amazon molly**. Muller's ratchet might cause extinctions of the unisexual Amazon molly due to the accumulation of slightly deleterious mutations. (**A**) analytical results only, (**B**) analytical results and simulations combined. The upper bar denotes the assumed age of the line (70 Ky, min 40 Ky, max 100 Ky). The lower bar marks the border to neutrality for the effective population sizes (*N*_*e*_) used by spanning the selection coefficients from *N*_*e*_*s *= 1 for the largest (10^7 ^females) to the smallest conceivable *N*_*e *_(10^4 ^females). The lines represent the analytic predictions of the extinction time for different deleterious genomic mutation rates (*U*_*sdm*_) with *N*_*e *_= 316,000, generation time *T*_*gen *_= 1 year and maximal reproductive capacity *R*_*max *_= 500 offspring/generation. Our current best overall estimate for *U*_*sdm *_is 0.4 critically deleterious mutations/diploid genome/generation. Our upper limit is close to *U*_*sdm *_= 1. The dashed lines indicate the variability of the extinction time estimates for a value that is close to our lowest credible mutation rate estimate (*U*_*sdm *_= 0.05, green diamantes) using the corresponding upper and lower limits of *N*_*e*_, *T*_*gen *_and *R*_*max*_; variability in extinction time is similar for other *U*_*sdm*_. Large symbols denote valid extinction time estimates from simulations with at least 2 observed clicks of Muller's ratchet (usually many more, up to 500). Small symbols denote lower limits for extinction times from simulations without observed clicks, based on the (usually wrong) assumption that the ratchet would have clicked just after stopping the simulation. Each symbol denotes an independent simulation with a different random seed and assumes the same mean *T*_*gen *_and *R*_*max *_as analytic predictions (different *N*_*e *_have been plotted on top of each other to avoid a series of similar plots). This plot contains 24,251 simulations with a total of 14.78 years of computing time. See main text for a discussion of parameter combinations and Loewe [7] for an in-depth explanation of the U-shaped plot shown here. The location of the *wall of background selection *for a particular parameter combination is approximately given by the vertical part of the corresponding line: all mutations with effects larger than the location of this wall are removed deterministically.

The results show that values for *U*_*sdm*_, the slightly deleterious mutation rate, that are above *U*_*sdm *_≈ 0.1, lead to the extinction of the Amazon molly within the estimated *T*_*age *_= 81,000 years of its existence, even if lower and upper limits for *T*_*age *_are considered (see Figure 1 and *T*_*age *_estimates below). This is also true for our best estimate of *U*_*sdm *_in the unlikely case of extremely high levels of mitotic recombination (then *U*_*sdm *_is scaled to 0.2 deleterious mutations with critical effects/diploid genome/generation). These findings are rather independent of the effective population size *N*_*e*_, as even *N*_*e *_= 10^7 ^(certainly larger than the true *N*_*e *_of the Amazon molly) will not help against Muller's ratchet if mutation rates are too high.

A deleterious mutation rate of about *U*_*sdm *_≈ 0.05 per generation results in extinction times that border with upper limits of estimates of the age of the Amazon molly of 10^5 ^years. At this mutation rate some values of *N*_*e*_, *R*_*max *_and *T*_*gen *_permit persistence and others cause extinction within this timeframe, where *R*_*max *_is the maximal reproductive capacity of the non-degenerated ancestors and *T*_*gen *_is the generation time. The variability due to *N*_*e*_, *R*_*max *_and *T*_*gen *_is indicated for *U*_*sdm *_= 0.05 by the dashed black lines in Figure 1. These factors lead to similar variability of extinction times for other mutation rates. If the deleterious genomic mutation rate were only 0.01, then extinction due to Muller's ratchet in the known age of the *P. formosa *lineage could be excluded, even with the current uncertainty in other parameters.

If deleterious mutation rates are greater than our best estimate of *U*_*sdm *_≈ 0.4, then Muller's ratchet will cause extinction of the Amazon molly in a few thousand years from the origin of asexuality under the standard model in the absence of mitigating forces. Such high mutation rates seem to be supported by our best approximation of the genetic architecture in the Amazon molly (many approximately codominant mutations of small effects in a genome with very low levels of mitotic recombination lead to an effective doubling of *U*_*sdm *_as shown in the Equal-Contribution-Model in Table 1 and discussed in the Models section below).

**Table 1:** Useful transformations for computing expectations and bounds for the rate of Muller's ratchet in diploids.

Genome type	Recessive (*h *= 0)	Co-dominant (*h *= 0.5)	Dominant (*h *= 1)
(1) asexual haploid	$\frac{U_{sdm}}{s}$	$\frac{U_{sdm}}{s}$	$\frac{U_{sdm}}{s}$
(2) asexual diploid	Core-Genome-Model (extreme forms are unrealistic) **Stage 1** (MA is easy, may be harmless): $\frac{2U_{sdm}}{sh}\to \frac{0}{sh}$ **Stage 2** (MA is harder, may be impossible): $\frac{0}{s\left(1-h\right)}\to \frac{<2U_{sdm}}{s\left(1-h\right)}$	Equal-Contribution-Model (useful first order approximation) $\frac{2U_{sdm}}{sh}$	Every-Allele-Needed-Model (most unrealistic) **Stage 1** (MA is hard, may be impossible): $\frac{2U_{sdm}}{sh}\to \frac{>>0}{sh}$ **Stage 2** (MA is easier, may be still hard): $\frac{0}{s\left(1-h\right)}\to \frac{<<2U_{sdm}}{s\left(1-h\right)}$
(3) asexual diploid with mitotic recombination	intermediate between genome type (2) and (4)	intermediate between genome type (2) and (4)	intermediate between genome type (2) and (4)
(4) automictic selfing diploid with free recombination	$\frac{2U_{sdm}}{2s}$	$2U_{sdm}/2s\frac{\left(1+sh\right)}{\left(1-sh\right)}$	$2U_{sdm}/2s\frac{\left(1+s\right)}{\left(1-s\right)}$

The table gives the variables in the exponent of $N_{0}=N_{e}\cdot e^{-U_{sdm}/s}$, where *N*_0 _is the number of individuals in the population that are in the 'best class' (has the highest fitness) in mutation-selection balance. Here we propose that Muller's ratchet in a given genome type can be approximated by using predictions for Muller's ratchet in a haploid asexual genome and applying the scaling given here. *U*_*sdm *_= slightly deleterious mutation rate/haploid genome, *s *= homozygous selection coefficient, *h *= dominance coefficient, *sh *= heterozygous selection coefficient, where in this table positive *s *denote harmful mutations. The two stages for asexual diploids denote the fixation of the first and second deleterious mutation that can occur at a diploid locus. For individual stages, arrows indicate the change of *U*_*sdm*_/*s *with increasing mutation accumulation (= MA). '<' or '>' indicate that mutation rates will remain below or above the indicated level, respectably.

Following Loewe [7], the threat of extinction from Muller's ratchet in Amazon molly can be quantified in detail as follows:

*U*_*sdm *_= 0.5 (and a mean of all other values) yields a minimal extinction time, *T*_*ex*_, of *T*_*ex *_≈ 5 Kyr due to most critical selection coefficients, *s*_*cm*_, in the range from *s*_*cm *_≈ 0.005 to 0.05. A genomic decay paradox for this mutation rate exists at *T*_*age *_= 100 Kyr in the range of critical selection coefficients *s*_*c *_≈ 0.0002 to 0.06.

*U*_*sdm *_= 0.1 (and a mean of all other values) yields a minimal extinction time of *T*_*ex *_≈ 50 Kyr due to most critical selection coefficients in the range from *s*_*cm *_≈ 0.005 to 0.01. A genomic decay paradox for this mutation rate exists at *T*_*age *_= 100 Kyr in the range of critical selection coefficients *s*_*c *_≈ 0.001 to 0.01.

Lower mutation rates under the same conditions lead to minimal extinction times that are longer than the assumed age of the line (*U*_*sdm *_= 0.05 leads to extinction in *T*_*ex *_≈ 200 Kyr at *s*_*cm *_≈ 0.005 and *U*_*sdm *_= 0.01 to *T*_*ex *_≈ 3 Myr at *s*_*cm *_≈ 0.001). See the coloured lines in Figure 1 for a visual overview.

If mutation rates are increased beyond the natural levels that we estimated (e.g. by mutagenic pollution), then the most damaging mutational effects are all in the range of several percent or more and resulting minimal extinction times can be surprisingly short. For example, *U*_*sdm *_= 1 can lead to extinction times of about 300 years by accumulating deleterious mutational effects of *s *≈ 10%. If mutagenic pollution leads to a further 10-fold increase of *U*_*sdm*_, then extinction times are expected to be less than 30 years, as increasingly harmful mutations start to accumulate as well (extrapolation from Figure 1).

## Discussion

This is the first detailed assessment of the threat of extinction from deleterious mutation accumulation through Muller's ratchet in the Amazon molly. Combining our best estimates of the haploid deleterious mutation rate (*U*_*sdm *_≈ 0.2) with our best approximation of the genetic architecture in the Amazon molly (many approximately codominant mutations of small effects in a genome with very low levels of mitotic recombination) leads to an effective deleterious mutation rate of *U*_*sdm *_≈ 0.4 mutations with critical effects/diploid genome/generation. In the absence of significant mitigating forces this would cause extinction of the Amazon molly in a few thousand years. More precise results can be taken from Figure 1 if needed. This represents a big step forward for understanding the asexuality in the Amazon molly, since never before have the times to extinction been quantified in such detail for this fish.

### Genomic decay paradox

Combining predicted extinction times with current estimates of the age of asexuality in the Amazon molly leads to a genomic decay paradox, as defined by Loewe [7]. Thus mechanisms that extend our standard model of Muller's ratchet are needed to explain why this fish has thus far escaped extinction. Such mechanisms are not needed if either our most plausible mutation rate estimates are too high or the Amazon molly is younger than current age estimates suggest (effective *U*_*sdm *_= 0.04 – 0.9; *T*_*age *_= 40,000 – 100,000 years, see section 'Age...' below and [11,12]). As current evidence seems hardly compatible with such low mutation rates or such a young age of *P. formosa*, the quest for mechanisms that help this fish escape genomic decay seems to be valid. This is corroborated by our observation of a fitness correlate, the number of embryos found in females, which does not show significant differences between *P. formosa *and *P. mexicana *(one of its parental species, see section on life history below). While we have no such information for other correlates such as longevity, number of broods per life, offspring survival, etc., our current limited evidence suggests that *P. formosa *experiences only little or no genomic decay despite our predictions of substantial deleterious mutation accumulation. Below we will discuss processes that might be of special importance for maintaining fitness in the Amazon molly.

### Mitotic recombination

Some reports of apomixis in the Amazon molly suggest a little debated potential solution to the mystery of its long-term survival. Rasch *et al. *[26] reported low, but consistent, levels of tissue graft rejections after prolonged periods (up to one year) within certain sibships, suggesting that not all inheritance is strictly isogenic in the Amazon molly. In the absence of a meiotic prophase these observations have been interpreted as the result of either a mutation rate that exceeds expectations or as the result of somatic cell crossing-over [26,32]. This process is also known as mitotic recombination and is most likely an inevitable result of the way that cells organize mitosis. It was first discovered in Drosophila [33,34] and has been intensively studied in yeast [35-37], mice [38], humans [39] and Daphnia [40]. The frequency of mitotic recombination in some fungi was found to be between 10^2 ^and 10^4 ^times less than that of meiotic recombination [41] and recent experiments in yeast reported a factor of ≈ 25,000 [37]. Estimates suggest that about 10 sister chromatid exchanges per cellcycle can occur in mammalian cells [42]. Such evidence suggests that the Amazon molly experiences mitotic recombination, even if the effective rate of segregation of different mutations is probably much lower than in selfers with meiotic recombination. The fact that asexual Daphnia have been shown to experience mitotic recombination [40] means that the Amazon molly would not be the only asexual to experience mitotic recombination.

If this is true, the resulting segregation might slow down Muller's ratchet for some selection coefficients [43-45], compared to expectations from the Equal-Contributions-Model described below. However, the fact that the distribution of mutational effects is expected to be very wide on a log scale [46,47] means that corresponding smaller selection coefficients will continue to drive Muller's ratchet. Thus it is difficult to see how mitotic recombination could stop the ratchet on its own without the contribution of other processes. We can use a simple model to put an upper limit on the maximal potential of mitotic recombination to stop Muller's ratchet. Mitotic recombination cannot possibly do more to stop fitness decay than in the case of completely free mitotic recombination. This allows for mutations to segregate at a maximal rate and has been used to model Muller's ratchet in selfers [45]. As shown below (see section on Muller's ratchet with selfing), a simple scaling of mutation rate and selection coefficient is enough to extend the standard model of Muller's ratchet to this case. Applying such a scaling to our results does not remove the genomic decay paradox that we find (see Table 1; it is questionable whether mitotic recombination will have a large effect, since the rates of mitotic recombination are probably far from free recombination). Similarly an analysis of the various levels of dominance that are possible for diploids and triploids as shown in Table 1 does not remove the paradox. Thus we will have to search for other solutions.

Rare recombination events during oogenesis in the Amazon molly are not conceptually different from mitotic recombination in the germ line or selfing. To limit the largest possible contribution of these processes towards stopping genomic decay we assume the most generous form of selfing, which is free recombination between both diploid copies. As our analysis above shows, the largest possible amount of recombination during oogenesis cannot stop Muller's ratchet – if no fresh genes are introduced by outcrossing of some sort.

### Paternal leakage

Occasional paternal leakage of fresh genetic material from sexual sister species could serve as a template for DNA repair [48] or restore genes that had been destroyed by Muller's ratchet [13,49]. Paternal leakage has been demonstrated to lead to the expression of paternal genes [13,50], suggesting a compensatory role in the Amazon molly [13]. There are two mechanistic scenarios that may facilitate this.

(i) Micro-chromosomes with a size of about 1% of the genome have been observed to leak from a sexual sister species to the Amazon molly [13,24]. If they carry an occasional random sample of genes from the non-degraded sexual genome into the degrading Amazon molly population, then the possibility exists that they might restore an ancient, non-degraded state of fitness. This could slow down Muller's ratchet enough to solve the genomic decay paradox [13,25]. Micro-chromosomes have been demonstrated to be stably inherited over many generations [25,51].

(ii) The finding of triploid clones might encourage the speculation that the third genome copy that is contributed by the sexual sister species might help restore fitness [49]. One might speculate that triploids should produce more offspring or survive better and ultimately substitute the diploid individuals. However, observations suggest the contrary, as triploids are rather limited in their range, young, and much less frequently produced than in other asexuals [15,23]. An alternative possibility is that triploids might occasionally lose one of their three genome copies and give rise to secondary diploids that can carry presumably fitter genes from their sexual sister species. If such a gene flow exists from the sexual sister species over paternal leakage and an intermediate triploid stage to a final diploid stage, then such a flow might contribute towards solving a genomic decay paradox [49]. However, there is no evidence for such a directed gene flow and triploids are produced much rarer in the Amazon molly than in other asexuals or than micro-chromosomes in *P. formosa *[23].

In any case, paternal leakage should not be confused with true recombination, which has stimulated discussions about the ratchet stopping potential of paternal leakage [52,53]. It has also been speculated that the paternal genome might be used as a template for DNA repair, but its precise role remains unclear [48].

### Other processes

There is a long list of other potential solutions for the genomic decay paradox that has been given elsewhere [7] and thus shall not be discussed in detail here. This list includes the unlikely possibility that the true deleterious mutation rate might be much lower, either because mutation rates in the Amazon molly happen to be generally much lower for yet unknown reasons, or because the distribution of mutational effects happens to be strongly bimodal with almost no mutations in the critical intermediate range. These hypotheses are not well supported by comparative analyses of mutation rates and effects in different species (see Parameter Estimates below). Synergistic epistatic effects have also been argued to have the capacity to stop genomic decay [54,55], but this is only true in combination with very specific distributions of mutational effects. If these distributions are reasonably wide, the potential for epistatic effects to decelerate Muller's ratchet is virtually non-existent [56].

We have also ignored advantageous and compensatory mutations, which have a substantial potential to stop genomic decay completely, if they are frequent enough [57-59]. Recent work has suggested that a substantial fraction of all mutations is advantageous [47,60]. If the underlying patterns are not caused by other processes and the selection coefficients of the corresponding mutations are large enough, then advantageous mutations can stop Muller's ratchet. Recent work in viruses has suggested that the ratio of beneficial to deleterious mutations can increase as the mean fitness decreases [61]. If similar dynamics hold for fish then Muller's ratchet may operate much slower, if at all. A more detailed discussion of back mutations and compensatory mutations can be found elsewhere ([7] and references therein).

To increase the precision of extinction time estimates it would be desirable to have more direct empirical estimates of mutation rates and effects in the Amazon molly. Such more precise estimates are needed when specific potential solutions for this genomic decay paradox are to be tested. The evidence presented here makes it seem unlikely that such added information will change our main conclusion that a genomic decay paradox exists for the current age estimates. For more details and additional potential solutions, please see [7].

### Red Queen

It is possible that Muller's ratchet is not the only process that leads to genomic decay and that the speed of Muller's ratchet may be significantly increased by other processes. The Amazon molly and its closely related sister species are known to harbor parasites [62,63]. These parasites probably decrease the fitness of their host substantially in the wild and co-evolve with it in an evolutionary arms race for survival. This scenario is described by the Red Queen hypothesis [3,5,64,65] and may in itself lead to the extinction of a species. A Red Queen scenario can also be caused by antagonistic co-evolution in general, which may occur in many circumstances, including evolving predator-prey or plant-herbivore relationships or intra-specific co-evolution. It is also known that the speed of mutation accumulation caused by Muller's ratchet is enhanced in a population that experiences Red Queen dynamics [6,66]. Empirical support for a prerequisite of the Red Queen hypothesis could be found in another member of the family Poeciliidae to which the Amazon molly belongs [67]. Thus the Amazon molly might participate in such an arms race, however we currently do not know the range of biologically realistic parameter combinations that are needed to quantify Red Queen dynamics here. The Red Queen Hypothesis predicts an increased load from parasites in asexuals, because they cannot adapt as fast as sexuals to newly evolving parasites. Based on this prediction one might not expect substantial Red Queen dynamics in the Amazon molly, since it seems to have about as many parasites as its sexual sister species [68]. Another attempt to discover Red Queen dynamics in the Amazon molly was also negative [69,70]. If the Amazon Molly is forced to constantly evolve as under Red Queen dynamics, then the genomic decay paradox might be more extreme.

### Implications for the origin of the Amazon molly

Many attempts to produce fertile asexual hybrids in the laboratory have been unsuccessful [71-73], but under natural conditions such hybridization attempts between the sympatric parental species of the Amazon molly, *P. mexicana *and *P. latipinna*, might happen occasionally. If such hybridization events occurred regularly, they could have led to a stable existence of the Amazon molly form, even though all individual hybrids are on their way to extinction as they will soon be replaced by fresh hybrids. In this case we expect multiple different young hybrid lines in random samples from the overall population of the Amazon molly, as each lineage of hybrids will be closer to its parental species than to other independent hybrids. The corresponding phylogenetic tree is expected to be polyphyletic.

Such a scenario is not supported by existing mtDNA data [12,72]. Amazon molly individuals sampled from a wide range of locations show a paraphyletic tree that is compatible with a single hybridization event in the distant past. This is based on the observation that Amazon molly mtDNA sequences cluster either with each other or with exactly the same ancestral sequence from the parental species [12]. If all sequences sampled in the future follow the same pattern, this rules out polyphyly and casts serious doubts over the hypothesis of multiple hybridizations in the past [12]. Combining this with the difficulties to produce asexual hybrids in the lab [71-73] suggests that the Amazon molly probably comes from a singularly rare hybridization success. This is in marked difference to some other species, where asexual hybrids are much easier to obtain [73]. Thus our quantification of Muller's ratchet only applies to the descendents of this one clone that appears to have an age of about 80 Ky.

We cannot distinguish whether the Amazon molly originated by a single ancient hybridization event or whether there was a small series of such events involving very similar parental individuals in the distant past. This is of no importance for our conclusions, as the subsequent course of mutation accumulation is not expected to be different from that in a population that goes back to a single hybridization event.

In order for other hybridization events to affect our conclusions, there would have to be a repeated production of fresh clones that could then constantly replace the decaying genomes in the population. This is expected to show in phylogenetic analyses. Current analyses show the absence of polyphyly and indicate that there is no ongoing hybridization in the wild [12,72]. Thus our analysis is appropriate for the time that came after the population was cut off from geneflow. This is the time *T*_*age*_that we estimate as the time that Muller's ratchet had for degrading the Amazon molly. In the case that future work uncovers multiple hybridization events, our analysis is valid for the lifespan of each clone, assuming no additional factors like interclonal competition that is independent of Muller's ratchet.

As current genetic data cannot infer what happened before the origin of our current lineage of the Amazon molly, this lineage might well be only the *last *unisexual Amazon molly that did not yet go extinct, with many others preceding it. As we know nothing about potential previous clones our ability to test the so-called "Frozen-Niche-Variation model" [73,74] is rather limited in this case.

### Conservation genetics

Since extinction time is very sensitive to changes in the mutation rate, it is conceivable that the anthropogenic release of mutagenic substances could lead to such a strong increase in mutation rate that extinction times are predicted to be in time frames that are frequently considered by conservation biologists. One of us (DKL) could actually observe a typical *Poeciliid *habitat that had lost almost all vertebrate life due to apparent water pollution (Altamira, Tamaulipas, Mexico, 2002). Although we have no evidence to decide whether this incident was mutagenic or not, pollution in general often has mutagenic side effects. This suggests the possibility of considerable pollution at least of parts of the habitat of the Amazon molly and would not be the first instance where pollution in rivers leads to a several-fold increase of mutation rates (see observations in ferns [75-78]).

### Quantifying Muller's ratchet in other ancient asexuals is easier now

It is well known that asexual lineages are typically short-lived. While this observation is central to theories about the origin of sex, it still needs to be properly quantified [1]. The evolution@home results used in this work make it manageable to quantify the threat from Muller's ratchet for the long list of other putatively ancient asexuals that have been suspected of being threatened by genomic decay [1,4,79,80], including the Amazon molly's close relative Poeciliopsis [74,81]. We suspect that it might not be possible to explain the existence of all these asexuals by the surprising discovery of a recent ancestor with a very young age as in the case of the clonal, hybrid, gynogenetic mole salamander Ambystoma [82-84]. Even if that were the case, then it would still be interesting to quantify Muller's ratchet, as this would shed more light on how fast it actually clicks in clonal lineages, where some evidence is consistent with its operation [85-89]. Examples of ancient asexuals that could benefit from a more rigorous quantification of the effects of Muller's ratchet include:

(i) *Darwinula stevensoni*, a small non-marine ostracod. Darwinulidae are believed to have lived for about 200 Myr without sex as the fossil record shows only females [80] – but see Smith *et al. *[90]. The species *Darwinula stevensoni *is a member of this group and is thought to exist for more than 20 Myr now [80,91,92].

(ii) Bdelloid rotifers. The Class Bdelloidea of the Phylum Rotifera is the largest taxonomic group that has apparently lived completely without sex for at least 40 Myr [93-95]. The ancient asexuality of these 0.1 to 1 mm long animals appears to be as well established as it can possibly be. Various special features of this group have been discussed as the reason behind its long-term survival [96-99].

(iii) Oribatid mites. Parthenogenetic automicts are frequent among oribatid mites [100,101]. Current estimates suggest that some lines have lived parthenogenetically for perhaps 100 Myr and that their extant distribution was strongly affected by continental drift [102].

#### Practical aspects

To quantify the possibilities of extinction from Muller's ratchet in a given asexual system using the evolution@home results database, please contact one of us (LL). You will receive help in completing a survey of various details about your study system (see online questionnaire [103]) and if enough data is available, a preliminary report will be produced (including plots similar to Figure 1). If necessary, the existing evolution@home infrastructure can be used to compute new parameter combinations. Our experience shows that the prediction of extinction times in ancient asexuals can often be simplified by using rough upper and lower limits for important parameters. For some parameters the lack of precision will not be critical, where as for others it will help focus further empirical work towards parameter estimation.

### Why quantify the ratchet as often as possible?

There are many open questions that can be asked about the general accuracy of the simple standard model of Muller's ratchet used for the quantifications presented here [7]. We believe that the following reasons justify a series of analyses of extinction times in various asexuals based on our null-model. We expect such work to contribute towards a mature, quantitative discussion about the evolutionary biology of asexuals.

(i) Critical predictions of extinction time help biologists to look for the key data that is also needed for more realistic quantifications of the effects of mutation accumulation. Therefore such predictions help in the design of empirical work.

(ii) It can be expected that at least some of the putative ancient asexuals are not examples of the genomic decay paradox, because the known age of their asexuality is smaller than their predicted extinction time. This does not deny the age of their asexuality, it just takes the 'scandal' out of the observations [4].

(iii) Those species with an apparent genomic decay paradox can be subject to a more detailed search for mechanisms that solve the paradox and help them to avoid extinction [7].

(iv) Experiences with the present simple system for predicting extinction times can be expected to lead to the development of more realistic systems for the quantification of genomic decay paradoxes. Such improved systems might include the processes discussed here and might measure their mitigating effects on mutation accumulation.

## Conclusion

A genomic decay paradox is predicted by a large number of biologically realistic parameter combinations for the unisexual Amazon molly. This is based on a simple model of Muller's ratchet that accounts for the distribution of mutational effects on fitness, the availability of multiple copies of the genome and mitotic recombination. Our prediction of a genomic decay paradox strengthens the conclusions of earlier work that suggested the existence of additional biological processes that slow down or halt the mutational decay of fitness in this fish. This conclusion is consistent with our observation that the Amazon molly carries approximately the same number of embryos per adult as its sexual sister species. Future work will have to establish whether paternal leakage of micro-chromosomes or still other processes have helped the Amazon molly to survive until today. If these mitigating processes are weak enough, an increase of mutagenic substances in the environment could easily lead to a rate of mutation accumulation that might allow extinction within time frames that are frequently of interest to conservation biologists.

## Methods

### Standard null model

To test the hypothesis that Muller's ratchet does not threaten the Amazon molly with extinction during the known time of its existence, we used the null model for quantifying the threat of extinction from Muller's ratchet, as described elsewhere in detail [7]. This model is a simple extension of the standard model of Haigh [104] combined with mutational meltdowns [105]. In short, we combined a multiplicative fitness model with an upper limit for *R*_*max*_, the maximal effective number of offspring that can be produced by an individual. This allows computing *C*_*mm*_, the number of clicks of Muller's ratchet that are needed to start mutational meltdown

(1) *C*_*mm *_= log(1/*R*_*max*_)/log(1 - *s*),

where each click is the stochastic extinction of the genotype carrying the fewest deleterious mutations that have the constant positive selection coefficient *s *[see [7,105]]. While this extinction does not require the fixation of a new mutation in the population, such a fixation typically follows shortly after the loss of the best class [106,107], unless there are special circumstances. Combining the effective population size *N*_*e *_with *U*_*sdm*_, the genomic rate of the origin of slightly deleterious mutations with effect *s*, allows us to compute *T*_*cl*_, the average effective time between two clicks of Muller's ratchet in generations that are assumed to be discrete. Since computation of this click time is rather difficult, we combine two of the best analytical approximations [9,27,28] with extensive individual-based computer simulations that were distributed over the Internet using evolution@home, the first global computing system for evolutionary biology [7,29-31]. We do not quantify mutational meltdown, because this demographic process at the end of genomic decay is so fast that it can be neglected. Therefore, we approximate *T*_*ex*_, the time to extinction by

(2) *T*_*ex *_= *C*_mm _* *T*_cl _* *T*_*gen*_

where *T*_*gen *_is the time between generations that are assumed to be discrete [7]. Extinction times are computed for a large number of parameter combinations that span the whole realistic range of parameters, to assess how many parameter combinations could lead to an extinction by *T*_*age*_, the known age of existence of the asexual lines of descent. Our simplifying assumption here is that no significant mutation accumulation had occurred in the sexual ancestors, since regular recombination would have facilitated the selective removal of all slightly deleterious mutations of critical effects (i.e. effects that could endanger the long-term survival of the Amazon molly in the presence of Muller's ratchet). To account for the distribution of mutational effects we scale the total genomic mutation rate appropriately to obtain *U*_*sdm *_[7] and we estimate *N*_*e *_from diversity data [108]. This approach is justified in the corresponding sections below.

### Mutation rate estimates

Before we can estimate *U*_*sdm*_, we need to estimate *U*_*tot*_, the mutation rate at all potentially deleterious sites in a haploid genome per generation. To do this we focus on synonymous point mutations for estimating the rate per base pair and later extrapolate to potentially harmful sites that change amino acids or affect regulatory functions. We focus on synonymous point mutations for several reasons.

(i) Synonymous mutations are expected to be mostly effectively neutral (or only under weak selection) and therefore their rate of fixation can be predicted from the neutral theory [109] (the rate is slightly lower if there is very weak selection). Thus synonymous substitution rates are probably very close to the true mutation rates of neighbouring non-synonymous sites that accumulate the actual mutational damage.

(ii) We cannot apply observations from microsatellites, since their mutational model is very different from that of normal point mutations and it is not clear, how an estimate of one rate could be converted to a direct estimate of the other rate.

(iii) One might want to ignore chromosome rearrangements and frame-shift mutations like indels or TE insertions, if one wants to obtain a conservative estimate of extinction time. Many of these mutations are so strongly selected against that they have no chance of accumulating in a large population (their selection coefficients are firmly behind the 'wall of background selection', see Figure 1 and [7]). Thus, such drastic mutations are probably only of importance in the context of corrections for polyploidy that were discussed above and if mutation rates are already very high.

In addition to mutation rates in the nuclear genome, one might want to consider mutation rates in the mitochondrial genome, since mitochondria are probably as essential to fish as they are to humans [7].

### Distribution of mutational effects

To quantify Muller's ratchet in the presence of a distribution of mutational effects on fitness we partition this distribution in three as suggested elsewhere [7,108]:

(i) Very deleterious mutations are implicitly dealt with by our method of determining *N*_*e*_, see below.

(ii) Slightly deleterious mutations with effects in the critical range or close to the critical range are the main focus of our attention here, as these contribute most to extinction, see below.

(iii) Effectively neutral mutations that accumulate like neutral mutations are ignored, as their effects are too small for impacting fitness.

In order to quantify Muller's ratchet we need a good estimate of *U*_*sdm*_, the slightly deleterious mutation rate, which is given by

(8) *U*_*sdm *_= *U*_tot _* *f*_*sdm*_,

where *U*_*tot *_is the genomic mutation rate at sites that are in a functional category with potentially deleterious effects like non-synonymous mutations, and *f*_*sdm *_is the fraction of sites with slightly deleterious, critical selection coefficients *s*_*c *_among all potentially deleterious mutations (for more details on this approach, see [7]). The corresponding range of critical selection coefficients approximates all *s *that lead to extinction within a minimal time or within a given time *T*_*age*_; this can be determined from Figure 1. To compute *f*_*sdm *_we need to combine the range of all *s*_*c *_with estimates of the distribution of mutational effects on fitness, which traditionally have been difficult to obtain. However recent progress has been substantial and we now know that the distribution of non-synonymous mutational effects is very leptokurtic and spans many orders of magnitude in many different species [46,47,110-112].

### Effective population size

There are three population sizes that are potentially relevant here:

(i) total census population size *N*_*t*_,

(ii) effective population size in the absence of any deleterious mutations *N*_*e*0_,

(iii) effective population size in the presence of background selection *N*_*eb*_.

Here we argue that *N*_*e *_= *N*_*eb *_for the purposes of quantifying the rate of Muller's ratchet; this is how we use *N*_*e *_outside of this section. In order to see this, we need to consider how to approximate the rate of Muller's ratchet in the presence of a wide distribution of mutational effects. New work by Söderberg & Berg [108] has shown that the rate of the ratchet in the presence of such a distribution can be approximated reasonably by dividing mutational effects into different categories (see above). Here we consider the category that contains mutations with very strong effects (see background selection theory [113,114]). Simulations [108] show that the effect of these mutations on the rate of the ratchet is well approximated by using a scaled effective population size *N*_*e*0 _that is appropriately reduced by the factor *N*_*eb*_/*N*_*e*0 _so that it only contains individuals that are free from strongly deleterious mutations (see equation 7 in [108]). This is consistent with classical Hill-Robertson effect theory, which states that a locus that is linked to another locus under selection will experience a reduction in effective population size [109].

In the absence of deleterious mutations, estimates of *N*_*e *_= *N*_*e*0 _can be made from diversity data and mutation rates, but we cannot estimate *N*_*e*0 _in our system, since deleterious mutations are present. Surprisingly, we do not need such an estimate, since background selection theory predicts that any estimate of *N*_*e *_based on diversity and mutation rates will in reality be an estimate of *N*_*eb*_, the effective size of the class that is free from strongly deleterious mutatons [113,114]. This means that two difficulties in our analysis cancel each other out: we do not need to estimate *N*_*e*0 _and we do not need to simulate background selection for a population of size *N*_*e*0 _to quantify the rate of the ratchet for mutations with critical effects. We only need to use standard approaches for measuring *N*_*e *_from DNA sequence diversity; this will approximately give us *N*_*eb *_for our simulations, which then have to ignore all mutations with effects in the background selection range [108]. Thus our analysis uses information from two categories of sites:

(i) Neutral sites are important for estimating *N*_*e *_= *N*_*eb *_under background selection, but not for the ratchet itself (ignore when computing *U*_*sdm*_).

(ii) Selected sites contribute towards operating the ratchet (use for computing *U*_*sdm*_), but cannot be used to estimate *N*_*e*_.

Since mtDNA and all nuclear chromosomes are completely linked with the same selective unit in the absence of outcrossing, *N*_*e *_is the same for both systems like in pure selfers [115,116].

### Simplifications overview

Here we approximate the effects of Muller's ratchet in a real diploid genome by proposing to choose (i) effective deleterious mutation rates that are adjusted for the corresponding ploidy level and exclude mutations with effects that are either too large to accumulate or too small to cause effective harm and (ii) effective selection coefficients that are adjusted for arbitrary levels of ploidy, dominance and mitotic recombination. The corresponding adjustments are compiled in Table 1 and explained below. Given the large degree of uncertainty about mutation rates and selection coefficients in our study organism, the errors from these approximations seem insignificant. Also, it is not possible to convincingly decide between the "Core-Genome-Model" and the alternative "Equal-Contribution-Model", although the latter seems to be much more realistic (both models are explained below). Therefore all possible options are explored by including a wide range of possible mutation rates and effects.

### Extensions for diploids and polyploids

To apply the model above to non-haploid organisms requires some adjustments to account for the fact that duplicate copies of genes can buffer deleterious effects. To avoid the complexity associated with recent models of the evolution of gene duplicates [117-121], we propose two simple models that allow the reduction of polyploids to an effectively haploid genome model: (i) The *Core-Genome-Model *assumes that one functional copy of each essential gene is sufficient and all other alleles can be discarded without problems, so that fitness degradation from Muller's ratchet can only start when the 'backup alleles' have been deactivated. (ii) The *Equal-Contribution-Model *assumes that each allelic copy contributes equally to fitness, so that selection coefficients are reduced and mutation rates are increased proportionally to the ploidy level. Both models are discussed below for diploid and triploid Amazon molly along with the impact of a variable dominance coefficient *h *on quantifications of Muller's ratchet.

### Core-Genome-Model

One might assume that only one copy of each important gene is really needed and that all additional copies from higher ploidy levels of the genome could be regarded as 'neutral backups' that can be deleted without any negative effect, as long as the last functional copy is still working. An extreme interpretation of this assumption implies that all deleterious mutations are completely recessive (dominance coefficient *h *= 0), which is rather unrealistic in many settings [122-130]. However, in less extreme cases, recessivity may be strong enough to reduce the effects of a complete gene knockout to effective selective neutrality (*N*_*e*_*sh *< 1). Under the Core-Genome-Model otherwise strongly deleterious mutations like indels or transposable element insertions can disrupt gene function in all additional copies and thus accumulate effectively like neutral mutations. Eventually the core genome will be distributed across all the different haploid copies of the original genome, as it is really only a combination of all the least damaged parts of the genome. Since one frame-shifting mutation is enough to transform a copy of gene *i *into a pseudogene, we can approximate its time to inactivation, *T*_*ko*_, by

(3) *T*_*ko *_≈ 1/(*l*_i _* μ_bp _* *f*_*ko*_)

where *f*_*ko *_is the factor that specifies the frequency of frame-shift or other knock-out mutations relative to μ_*bp*_, the synonymous point mutation rate/basepair/generation, and *l*_*i *_is the total length of gene *i*. Lets assume a typical gene has 2,000 base pairs and frame-shifts occur at a rate of μ_bp _* *f*_*ko *_≈ 1*10^-9 ^(probably a lower limit if compared to ≈ 1*10^-8 ^mutations/bp/generation in mice and *f*_*ko *_≈ 1/3 as observed in bacteria [see the 'C' parameter in [131,132]]). In this case one may have to wait on average for about 500,000 generations, before inactivation of such a gene can be expected. Since these events most probably follow a Poisson distribution, it can be expected that a substantial fraction of essential genes would be inactivated very quickly, leaving only one copy that is then maintained by selection. It is these last copies that are then slowly degraded by Muller's ratchet, since they can most probably mutate to such slightly deleterious states that allow the operation of the ratchet. Early inactivation of enough 'backup copies' will give Muller's ratchet extensive periods of time for degrading the core genome (late inactivation may allow for slightly deleterious point mutations in the non-deactivated allele). For example, the rates above suggest that frame-shift accumulation in 20,000 diploid genes would inactivate on average about 1,000 genes (sd = 32) over 25,000 generations. A precise calculation of extinction time in this model would require the integration of the effects of increasing mutation accumulation over time, where the increase is caused by rising deleterious mutation rates due to the progressive inactivation of genes. To avoid these complex calculations, the following two extreme simplifications are proposed.

A lower limit of *T*_*ex *_can be obtained by ignoring all additional copies of the essential core genes. In this case the resulting *U*_*sdm *_to use for the computation of click time is that of the haploid core copy of the genome. An upper limit can be obtained by partitioning time into (i) the accumulation of knockouts while ignoring Muller's ratchet and (ii) the operation of Muller's ratchet while neglecting any further increase of mutation rates due to additional knockouts. Testing several possible ratios of these two times allows minimization of extinction time between the extreme of no time for frameshifts (no extinction due to Muller's ratchet, since mutation rates are too low) and all time for frame-shifts (no extinction, because Muller's ratchet will always need some time to cause an extinction, even with high mutation rates).

In the case of triploids or higher ploidy levels, the Core-Genome-Model needs correspondingly longer for genomic decay, but in no case can additional copies stop genomic decay. Higher ploidy levels are not discussed here, since we assume that the first Amazon molly was diploid. Triploids lose their advantage in the Core-Genome-Model, if they occasionally lose one of their three sets of chromosomes, as they might do [49]. The fact that this is possible indicates that the lost set of chromosomes does not carry any last functional copy of a vital gene. For more details on triploids see the Equal-Contribution-Model.

### Equal-Contribution-Model

#### Diploids

Many mutations of small effects do not seem to be completely recessive, but rather close to codominance, especially if their effects are very small [122-130]. If that is approximately true for most genes, then both copies in a diploid genome are actually needed to produce the required dose of proteins to maintain fitness. In this case, all copies of the genome of the Amazon molly will contribute approximately the same amount of functionality, implying that the size of the mutational target increases two-fold, while effective selection coefficients decrease two-fold, relative to the haploid case (there are twice as many sites to hit and selection only operates against heterozygotes with strength *sh*, where *h *= 0.5).

#### Triploids

It is not clear whether such reasoning can be extended to triploids, as ancestral genomes may have been fine-tuned for diploidy. The fact that triploids can have significant disadvantages if compared directly to diploids [133,134] cautions against a positive functional role of the additional copy of the genome. On the other hand, one could argue that the surprising flexibility of fish in tolerating various ploidy levels [133] allows for an easy incorporation of an additional dosage of proteins provided by a third set of chromosomes. This is not contradicted by the fact that Amazon molly triploids can be observed in the wild [23], as strong purifying selection would predict vanishing frequencies, since the rates of origin of triploids are rather low in this fish [23]. Existing data on expression patterns of muscle proteins in Amazon molly triploids are inconclusive [50]. These complications suggest that triploids may face purifying selection and therefore they are not likely to reach 100% in a population. Depending on the specific mechanism of how triploids incur a possible disadvantage, the inactivation of one copy might occasionally even be beneficial for triploids. In addition, triploids may revert back to diploidy by occasionally losing one of their three sets of chromosomes [49]. The fraction of triploids in the wild seems to fluctuate substantially, but no population with 100% triploids has been observed. Frequencies of triploids for two different samples were in the range of 4%–15% (Lamatsch et al., unpublished) and 3% – 46% [135], indicating that selection against triploids is probably weak. Therefore, equal-contribution-corrections to haploid estimates of *U*_*sdm *_(and *s *if available) have to be treated with caution in the case of triploids, but appear to be reasonable approximations for diploids.

### Varying dominance

The two models above assume either complete recessivity or codominance. Observed dominance levels in other species are frequently somewhere in-between, where mutations with smaller effects tend to be closer to codominance [122-130]. While we know very little about the precise values of the dominance coefficient, *h*, and the selection coefficient, *s*, in the Amazon molly, it appears to be highly probable that general findings from other species apply here too. Of special interest here is the fact that the distribution of mutational effects on fitness appears to be very wide on a log scale [46,47]. Thus we can propose a simplification that allows for arbitrary dominance coefficients between *h *= 0 and *h *= 1 by making slight adjustments to the effective *s *used in simulations. The simplification is based on the observation that

(i) deleterious mutations with smaller effects have a higher probability of accumulating in the population than those with larger effects and

(ii) mutations with large enough effects do not accumulate (see the 'wall of background selection' in Figure 1 and in [7]).

To simplify the treatment we speak of the ratchet as if it fixes mutations, although strictly speaking the ratchet will only cause the mutation-free class to go extinct and fixing happens shortly after that by genetic drift (see [106,107]). The following qualitative analysis is supported by published simulation results [106].

We can treat *dominant *mutations as follows. If and only if the ratchet can fix the first mutation at a site (i.e. individuals that are homozygous for that size go extinct and all individuals in the population become heterozygous) then it will eventually also fix the second mutation (i.e. all individuals become homozygous for the deleterious allele). If a heterozygote cannot be fixed, there is no way the homozygote state can be fixed and the effective mutation rate used to compute the speed of the ratchet should be reduced accordingly to exclude such sites.

In the case of *recessive *deleterious mutations, there are the two following extremes. If *s *is small (most mutations), then click times are hardly affected and almost no correction is necessary. If *s *is large enough to prevent fixation of heterozygotes, then again the effective mutation rate may be reduced, as fixation of the homozygous state is impossible. Only few intermediate *s *can become heterozygous but not homozygous, as they have to be close to the switch-like transition between 'can accumulate' and 'cannot accumulate' (see the 'wall of background selection' in Figure 1 and [7]) and their homozygous effects have to be too deleterious to accumulate. This simplification relies on the almost switch-like transition between mutation accumulation by Muller's ratchet and complete mutation removal by background selection.

We ignore *overdominant *sites, assuming that these stay in their optimal heterozygous state. This will lead to a conservative estimate of extinction time, as these sites do not contribute to fitness decay under this assumption. Future models will have to investigate to what extent such an approximation is justified in the presence of a small fraction of overdominant sites [129,130].

### Extensions for mitotic recombination

Non-meiotic recombination can play a substantial role in reducing diversity in asexual lineages [40]. To assess the extent to which the rate of Muller's ratchet could be reduced by this process [43,44,136], the following approach was used. We assume that the maximal possible rate of mitotic recombination is equivalent to selfing with free recombination between loci. The true reduction of the rate of Muller's ratchet is probably much smaller, since mitotic recombination is certainly not as effective as free recombination in a selfing population. Thus the strongest possible reduction of the rate of Muller's ratchet from mitotic recombination can be estimated from the analytical work of Heller and Maynard Smith [45]. As explained below, a simple scaling of *U *and *s *is enough to reduce predictions to the haploid case.

### Muller's ratchet with selfing

Heller and Maynard Smith [45] derived an equation that describes the distribution of deleterious mutations in a selfing population under Muller's ratchet. We assume here that the expectation of the size of the 'best class' in mutation-selection balance mainly determines the rate of Muller's ratchet. This appears to be supported by some simulation results [43,44] although Gordo & Charlesworth [137] found that the ratchet could click at different rates for the same size of the best class if other parameters were different. Our assumption allows us to use a simple scaling of *U *and *s *to reduce predictions for diploid selfers to the haploid case.

In detail, Heller and Maynard Smith [45] show that in a selfing population *x*_*ij*_, the fraction of individuals carrying *i *homozygous and *j *heterozygous mutations is given by

(4) $$x_{ij}=\frac{1}{i!}{\left[\frac{U_{sdm,d}\left(1-sh\right)}{2s\left(1+sh\right)}\right]}^{i}\cdot \exp\left[-\frac{U_{sdm,d}\left(1-sh\right)}{2s\left(1+sh\right)}\right]\cdot \frac{1}{j!}{\left[\frac{2U_{sdm,d}}{1+sh}\right]}^{j}\cdot \exp\left[-\frac{2U_{sdm,d}}{1+sh}\right],$$

where *U*_*sdm*, *d *_is the slightly deleterious mutation rate/diploid genome/generation that changes wildtype homozygotes into heterozygotes, *h *is the dominance coefficient and *s *is the homozygous selection coefficient (positive for harmful mutations). This equation assumes that (i) recombination is free between sites, so that the ratchet only operates at homozygous sites, since selfing can restore the best class on a heterozygous site, (ii) *U*_*sdm*, *d *_is constant and independent from the number of mutations per individual, (iii) mutations at heterozygous sites are negligible, since selfing produces many more homozygotes, (iv) the standard model of Muller's ratchet without back mutations is valid [7,104]. Then the number of individuals in the 'best class', *N*_0_, can be computed by adding up all heterozygotes that are free from homozygous deleterious mutations and then scaling by the effective population size

(5) $$N_{0}=N_{e}\cdot \sum_{j=0}^{\infty }x_{0j}$$

Using the well known result that

(6) $$\sum_{j=0}^{\infty }\left({\frac{x}{j!}}^{j}\right)=\exp\left(x\right)$$

equations (4) – (6) yield

(7) $$N_{0}=N_{e}\cdot \exp\left[-\frac{U_{sdm,d}\left(1-sh\right)}{2s\left(1+sh\right)}\right].$$

For complete recessivity (*h *= 0) this reduces to the haploid asexual case ($N_{0}=N_{e}\cdot e^{-U_{sdm}/s}$), because the diploid mutation rate *U*_*sdm*, *d *_equals twice the haploid rate *U*_*sdm*_. It is easy to see from (7) that the degree of dominance of mutations with small selection coefficients does not significantly affect numerical values and hence the rate of the ratchet. Since the ratchet does not click for large *s*, one might as well neglect dominance altogether.

## Parameter estimates

### Effective population size

For our simulations we need an estimate of the effective population size in the presence of background selection (*N*_*e *_= *N*_*eb*_, see above). To arrive at such an estimate we obtain corresponding diversity data from the control region of mtDNA, since it is linked to the rest of the genome by unisexual inheritance (see the Models section above for a justification of this approach). We use the DNA sequence diversity data from Möller (see pp. 46, 47 and 55 in [12]), who observed 14 haplotypes with *S *= 13 sites segregating for single base pair polymorphisms in a total of *n *= 63 sequences of length *L *= 886 bp from 17 geographic locations. Since the control region of mtDNA is likely to evolve neutrally at most sites, we can use Watterson's [138] equation to compute *N*_*e *_= *S*/(L * μ_bp _* *a*), where *N*_*e *_is the effective population size of mitochondrial DNA in the presence of background selection, μ_*bp *_is the mutation rate per site/generation and *a *= 1 + 1/2 + 1/3 + ... + 1/(*n*-1) for *n *sequences in the sample. Here we assume an mtDNA divergence rate of 3.6% ± 0.46%/site/Myr as estimated in the species groups of the fish *Centropomus *[139] from divergence since the Panama seaway closed 3.0–3.5 Myr ago [140]. This suggests a value for *N*_*e *_between 153,000 and 198,000 that will have to be scaled downward, if actual mutation rates are higher, like in human pedigrees [141]. The nuclear value is identical to this mtDNA estimate, if there is no outcrossing [115,116].

Comparative analyses suggest that the resulting values are reasonable. The total population size *N*_*t *_may be used to place upper bounds on *N*_*e *_in most situations. Some mark and recapture experiments could only estimate that *N*_*t *_> 10,000 in that local study area (M. Doebler, personal communication). One may speculate that about 10 million individuals of *P. formosa *may exist in total. If this is combined with the typical finding that vertebrates frequently have *N*_*e*_-values that are about 10% of their census population sizes [142], then *N*_*e *_would be about a million. Given that a species as abundant as the fruitfly *Drosophila melanogaster *has effective population sizes of little more than a million [143], suggests that the Amazon molly probably has *N*_*e *_< 10^6^. On the other extreme it is highly unlikely that the Amazon molly would have an effective population size smaller than that of primates, suggesting *N*_*e *_> 10^4^.

We use deliberately wide upper and lower bounds of *N*_*e *_= 10^4 ^to 10^7 ^to demonstrate that population size has a rather small influence on the operation of Muller's ratchet in the presence of a distribution of mutational effects.

### Life history

Based on observations we estimate that generation time *T*_*gen *_is between 4 month (min) and 2.5 years (max), so we assume 1 year as our middle value. The maximal reproductive capacity in the wild with a non-degraded genome *R*_*max *_is assumed to be 50 (min) 500 (mid) and 2000 (max) offspring per lifetime (see below). While *T*_*gen *_scales extinction times linearly, *R*_*max *_has a much smaller effect. It is possible that our values for *R*_*max *_are too large since they are more based on the highest possible values conceivable under laboratory conditions than on any real life survival situation. Once such data are available, results from Figure 1 can be easily scaled appropriately (see equation 2).

Unfortunately there is very little information about life history from natural habitats. Most of the knowledge about *P. formosa *comes from extensive aquarium cultures. In captivity the Amazon molly reaches maturity between 4 and 6 months, produces more offspring at larger body sizes and may live for up to 3 years under optimal laboratory conditions. Here we report that females caught in the wild contain a mean of 28 embryos (sd ± 16; *n *= 20; observed maximum in the wild = 70 embryos, slightly less than the maximum of 90 observed in the lab [144]). Females will probably produce this number of newborn fish about every 28 days, depending on water temperature. They stop reproduction about 3 to 6 months before death in the lab. The number of surviving newborn fish can be reduced considerably by predation or disease. Thus under good conditions *R*_*max *_might be 18 periods * 28 offspring = 504, assuming a life span of 2 years. As a lower limit one might use 50 offspring per lifetime, assuming that many offspring die due to predation or disease and many parents are small and frequently trapped in little ponds without sister species that provide the sperm that they need as a trigger for development. An upper limit of *R*_*max *_= 2,030 might be derived from combining the maximal number of offspring-producing periods observed in the lab (29 = 36-4-3) with the maximal number of embryos per period observed in the wild (70 in *n *= 20, the upper expectation for a fish of this size [10]). We did not find that the sexual sister species, *P. mexicana limantouri *differs significantly from these values (observed mean = 27 embryos ± sd = 13; *n *= 11).

### Age of the evolutionary line

The ancestor of the Amazon molly clone known to us was formed by a hybridisation event between a *Poecilia mexicana *female and a *Poecilia latipinna *male [11,12] and is believed to have reproduced asexually since then. A date *T*_*age *_for this event may be derived from comparing alleles in the Amazon molly with alleles in their corresponding parent species using a molecular clock. At the moment there are two datasets that are large enough to infer an evolutionary age that is different from zero. Comparisons of 1,377 bp in various nuclear genes (mostly introns) lead to a point estimate of *T*_*age *_= 100,000 years assuming a divergence rate of 2%/Myr from standard mitochondrial clocks [11]. This age may be an underestimate, since nuclear mutation rates are usually much lower than mitochondrial rates. However, the confidence intervals for this estimate range from *T*_*age *_= 8,930 to *T*_*age *_= 209,000 years due to the small number of substitutions observed [69,70]. Thus it is desirable to arrive at a more precise estimate. This can be done using the same set of mtDNA diversity data that was used to estimate *N*_*e *_above [12]. Analyses of the median joining network show that there is only one mtDNA haplotype that is shared between *P. formosa *and *P. mexicana *and that this haplotype is at the center of *P. formosa *diversity (p.49 in [12]). Thus it is reasonable to assume that this haplotype was the most recent common ancestor (MRCA) of all Amazon mollies and one can use the number of mutational steps back to the MRCA as an indicator of the time since divergence of Amazon mollies from their MRCA. Using the method described by Saillard et al. [145] and the data of Möller [see p.69 [12]] leads to an estimate of 1.294 (± sd = 0.159) mutational steps back to the MRCA in the 886 bp of the control region of mtDNA that were sequenced for this purpose. If this is combined with the divergence rate of 3.6% ± 0.46%/site/Myr as estimated in the species groups of the fish *Centropomus *[139] from divergence since the Panama seaway closed 3.0–3.5 Myr ago [140], then we arrive at an estimate of *T*_*age *_= 81,000 years for the time to the MRCA of *P. formosa *(1.294/(886 * 3.6 *10^-8^/2); lower and upper limits range from 63,000 to 104,000 years). This time will have to be scaled downward, if actual mutation rates are higher, as has been found in human pedigrees [141]. Thus we have marked *T*_*age *_= 40 Kyr, 70 Kyr and 100 Kyr as estimates of the age of the Amazon molly in Figure 1. It is easy to check our conclusions, if more precise information becomes available. A more rigorous analysis involving multiple genes from multiple individuals from each species would be desirable [see [146]].

### Mutation rate estimates

The last decades have seen a large increase of our understanding of mutation rates across a wide range of organisms [132,147,148] and we have no reason to assume hat the Amazon molly might have extraordinarily low mutation rates. Thus it is possible in the absence of more specific data to extrapolate from other species to arrive at a credible estimate for the Amazon molly specific mutation rate at all potentially deleterious sites in a haploid genome per generation, *U*_*tot*_.

The size of a haploid genome is about 950 Mbp, as derived from DNA content of cells [149]. If this is combined with the mutation rate counterpart of the divergence rate that was used to date the age of Amazon molly [11], then about 9.5 new mutations per haploid genome can be expected in each new generation (950 Mbp * 0.01 subst/bp/Myr assuming *T*_*gen *_= 1 year). Since many of these mutations will be synonymous or affect only non-functional DNA, we have to scale this rate by the effective genome size [132]. Fish are presumably more complex than flies. Therefore, we may use the effective genome size of *Drosophila melanogaster *as a lower limit (13,379 genes with 54,934 exons and a total length of 27.8 Mbp in exons [150] suggest about 19 Mbp as the target size for non-synonymous, non-frameshifting mutations). This is just 2% of the actual genome size and has to be increased to about 8% to account for non-coding functional sequences that seem to be about three times as abundant as functional sequences in exons in organisms as diverse as fruitflies and mice [151,152]. This would result in *U*_*tot *_≈ 0.74 potentially deleterious mutations/haploid genome/generation and may be an underestimate, as the amount of coding DNA in fish might be higher than in Drosophila. If a similar calculation is based on the 33 Mbp exons that we can estimate from the Fugu genome project, then *U*_*tot *_≈ 0.88 seems plausible (all genes occupy 108 Mbp and we estimate that this includes roughly 75 Mbp introns from Figure 2 in [153]).

These estimates do not change much, even if we use a completely different way of obtaining the mutation rate. Observations in mice have lead to estimates of 1.8 × 10^-10^/site/cell replication, respectively [132]. If an estimated 88 Mbp functional sites in *P. formosa *experience about 25 germ line cell divisions per adult generation (as female mice [132] that have a similar body weight to the Amazon molly) then we arrive at *U*_*tot *_≈ 0.4 potentially deleterious mutations/haploid genome/generation. Obviously, *P. formosa *is not a mouse and there is much room for improving the precision of such mutation rate estimates, even in mice. Recent high-precision measurements of the deleterious mutation rate in mutation accumulation experiments in Drosophila found *U*_*tot *_≈ 0.6 deleterious mutations/haploid genome/generation [154]. Scaling this estimate by the relative effective genome size of Fugu suggests *U*_*tot *_≈ 0.7.

It is encouraging to find such general agreement between these estimates that are based on very different approaches. This suggests that we can have some confidence in our estimates, even in the absence of direct observations in the Amazon molly. Since mutations are the inevitable consequence of DNA replication errors and the number of mitotic cell divisions per generation is a key determinant of mutation rates per generation [132,155], we conclude that the absence of recombination in the Amazon molly is unlikely to cause a major change in mutation rate when compared to its sexual sister species.

### Distribution of mutational effects

Recent progress has shown that the distribution of non-synonymous mutational effects is very leptokurtic and spans many orders of magnitude in many different species [46,47,110-112]. While we have no specific data for the Amazon molly, we have no reason to believe that this result does not hold here as well. One can use data on the ratio of non-synonymous to synonymous substitution rates, *K*_*A*_/*K*_*S*_, to infer that the fraction 1-*K*_*A*_/*K*_*S *_of all mutations must be more deleterious than about *s *= 1/*N*_*e*_, since mutations with effects below that threshold accumulate as if they were neutral [47,156]. If the data in Drosophila can be used as a landmark in this new territory, then about 90% of all mutations are more deleterious than the limit of 1/*N*_*e *_≈ 10^-6 ^[112]. Estimates of the distribution of mutational effects in Drosophila have shown that this distribution may well follow a lognormal law and there may be good theoretical reasons for this [46]. Robust features are its large width on a logscale and the fact that most probability mass is between effective neutrality and lethality [46,47]. From this and from Figure 1 we may infer that the fraction of slightly deleterious mutations, *f*_*sdm*_, could be perhaps around 30%, probably larger than 10%, but probably not much larger than 50%. We use these values as a point estimate with lower and upper bound. Thus *U*_*sdm *_probably shares the same order of magnitude with *U*_*tot*_. As this calculation has large errors, an array of other mutation rates is included as well when quantifying the ratchet. We assume that selection coefficients did not change much for most genes in the time since asexuality arose in the Amazon molly. This is compatible with the assumption that most genes in the Amazon molly are generic to similar fish species and thus well adapted, while only a small fraction is actually responsible for the specific adaptations of the Amazon molly.

### Deleterious mutation rates summary

As discussed above, our best estimate for the total mutation rate at potentially deleterious sites per haploid genome per generation is expected to be between *U*_*tot *_≈ 0.4 and *U*_*tot *_≈ 0.9, while our best estimate of the fraction of mutations with critical slightly deleterious effects is expected to be between *f*_*sdm *_≈ 10% and *f*_*sdm *_≈ 50%. Combining these values makes us expect *U*_*sdm *_≈ 0.2 slightly deleterious mutations with critical effects/haploid genome/generation with a lower limit of *U*_*sdm *_≈ 0.04 and an upper limit of *U*_*sdm *_≈ 0.45. These values may have to be doubled, depending on the genome model that is used for accounting for the effects of diploidy. To err on the side of caution and provide a better feeling for the effects of mutation rates we plot values from *U*_*sdm *_= 0.01 to *U*_*sdm *_= 1 in Figure 1.

## Authors' contributions

LL conceived the study, wrote the software, managed the computation of simulation results, developed the theoretical aspects of the paper, estimated many parameters, analyzed the results and wrote the manuscript. DKL provided most details on the Amazon molly biology, contributed to parameter estimation and helped writing the manuscript. Both authors read and approved the final manuscript.
